# A Brief View of the Surface Membrane Proteins from* Trypanosoma cruzi*

**DOI:** 10.1155/2017/3751403

**Published:** 2017-06-05

**Authors:** Ángel de la Cruz Pech-Canul, Victor Monteón, Rosa-Lidia Solís-Oviedo

**Affiliations:** ^1^Centre for Biomolecular Sciences, The University of Nottingham, University Park, University Blvd, Nottingham NG7 2RD, UK; ^2^Investigaciones Biomédicas, Universidad Autónoma de Campeche, Av. Patricio Trueba s/n, Col. Lindavista, 24039 Campeche, CAM, Mexico

## Abstract

*Trypanosoma cruzi* is the causal agent of Chagas' disease which affects millions of people around the world mostly in Central and South America.* T. cruzi* expresses a wide variety of proteins on its surface membrane which has an important role in the biology of these parasites. Surface molecules of the parasites are the result of the environment to which the parasites are exposed during their life cycle. Hence,* T. cruzi* displays several modifications when they move from one host to another. Due to the complexity of this parasite's cell surface, this review presents some membrane proteins organized as large families, as they are the most abundant and/or relevant throughout the* T. cruzi* membrane.

## 1. Introduction

**Figure 1:**
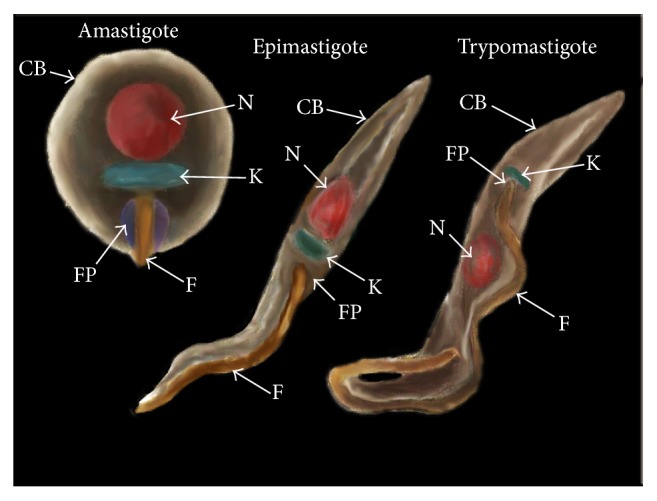
The different stages of* Trypanosoma cruzi*. The image depicted the amastigote, epimastigote, and trypomastigote stages from* T. cruzi* and their membrane domains: nucleus (N); kinetoplast (K); flagellum (F); flagellar pocket (FP); and cell body (CB).

*Trypanosoma cruzi* is a protozoan causative of Chagas' disease, a pathology characterized by two phases: acute and chronic; both could be asymptomatic. The acute phase is present during the first weeks of infection and the chronic phase includes an indeterminate asymptomatic form and a chronic inflammation associated with myocarditis, heart failure, and megaviscera (megaesophagus and/or megacolon) [1, 2].* T. cruzi* has infected millions of people in the world, mostly in Central and South America; the infection could be via triatomine insect vector, congenital transmission, organ transplantation, or blood transfusion [3, 4]. The life cycle of* T. cruzi* comprises several morphological transformations involving both mammalian and insect hosts, where three different major developmental stages are identified: epimastigotes, trypomastigotes, and amastigotes (Figure 1) [5, 6]. The developmental stages of* T. cruzi* alternate between noninfective and infective forms. Epimastigote and amastigote are noninfective but replicative stages in the gut of the insect vector and inside the mammalian cell, respectively. Trypomastigote stage is infective but nonreplicative and can be also considered as two different developmental stages: the bloodstream trypomastigotes, found in the blood of the vertebrate host, and the metacyclic trypomastigotes, found in the rectum of the insect vector [6–9]. If one considers that the cycle starts with insect sucking the blood of mammalian host infected with the bloodstream trypomastigotes, the ingested trypomastigotes transform into epimastigotes inside the insect stomach and replicate intensely in the midgut. After that, metacyclic trypomastigotes arise from epimastigotes in the hindgut of the insect host which are eliminated with the faeces [6, 10]. When the insect vector takes a blood meal from a new noninfected host it subsequently defecates in the area near the puncture wound. The infection usually takes place through direct inoculation of excreted metacyclic trypomastigote which forms into the lesioned skin caused by the insect vector bite. Once inside the mammalian host, the metacyclic trypomastigote forms invade the host cells at the inoculation site and transform into the replicative amastigote form. Upon completion of a replicative cycle as intracellular amastigotes, they transform back into bloodstream trypomastigote forms which burst the eukaryotic cell host and are capable of infecting other cells or travel into the bloodstream [11]. The phenotypic and genotypic diversity of* T. cruzi* are well recognized.* T. cruzi* is partitioned into six discrete typing units (DTUs), TcI–TcVI. For a comprehensive review see [12, 13]. Despite the fact that many* T. cruzi* isolates have been described through the years, CL Brener was the reference organism used in the “*Trypanosoma cruzi* Genome Project.” CL Brener is a clone derived from CL strain belonging to Tc VI lineage and has been thoroughly studied and well characterized [14–16]. The CL Brener genome sequence is now available and became the* T. cruzi* genome reference for other sequencing projects [13, 14, 17]. Moreover, new* T. cruzi* isolates are still being reported and features such as some of their surface membrane proteins are regularly compared with the CL Brener genome [17–20]. Although* T. cruzi* has several morphological transformations through its complex life cycle, studies on surface proteins have been mainly focused on the different stages through the infection process (Figure 1) [21–23]. Membrane proteins have been shown to play an important role in the biology of* T. cruzi*, including the interaction between parasite and host [2, 22, 24–28]. Scientists have made efforts to unravel the gaps on the structure and functions of these surface membrane proteins. However, despite their importance, the information is currently scattered. The aim of this review is to outline the families of surface membrane proteins from* T. cruzi* which are the most abundant and/or relevant during its life cycle (Table 1).

## 2. Mucin Family

**Figure 2:**
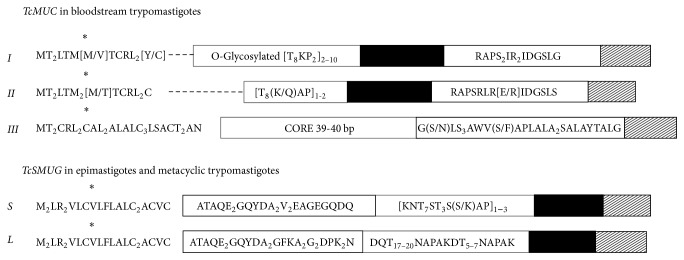
Mucin family. Schematic representation of mature proteins of mucin families TcMUC, found in bloodstream trypomastigotes, and TcSMUG, found in epimastigotes and metacyclic trypomastigotes. Signal peptide (*∗*); protein fingerprints (white boxes); hypervariable region (---); threonine-rich region (black boxes) and glycosylphosphatidylinositol- (GPI-) anchor signal (shadowed boxes). Image based on Buscaglia and Frasch [30, 60].

*Trypanosoma cruzi* is covered by a dense layer of mucin-type molecules. Mucins are the major* T. cruzi* surface glycoproteins and their sugar residues are able to interact with mammalian cells [26, 63]. These proteins are characteristic and widely distributed over the cell body, flagellum, and flagellar pocket of the different developmental forms (Figure 1) [64]. Mucins play a key role in the parasite protection as well as in the infectivity and modulation of the host immune response throughout the* T. cruzi* life cycle [25, 30, 60, 65, 66]. Based on sequence comparisons,* T. cruzi* mucins have been split into two gene families, termed TcMUC and TcSMUG (Figure 2, Table 1) [30, 67]. TcMUC expression seems to be restricted to the mammal-dwelling stages; these proteins are divided into three groups based on their central domains: TcMUC I to III [67, 68]. TcMUC I and II proteins are distributed on the amastigote and the bloodstream trypomastigote surface. TcMUC I is the major component in the amastigote form, whereas TcMUC II is predominant in membrane lipid rafts of the trypomastigote stage [31]. TcMUC I proteins show internal tandem repeats on their structure with a T_8_KP_2_ amino acid (aa) consensus sequence which are suitable targets for the O-glycosylation pathway in* T. cruzi*, flanked by an N-terminal signal peptide and a C-terminal glycosylphosphatidylinositol- (GPI-) anchor signal [30, 69]. TcMUC II genes encode proteins that share similar N- and C-termini with TcMUC I but without the T_8_KP_2_ motifs, although their central regions are still rich enough in threonine, serine, and proline residues [29, 70]. The single gene product of the TcMUC III group is termed trypomastigote small surface antigen (TSSA) and has been identified as a mucin-like glycoprotein (tGPI-mucins) [71]. TSSA are displayed on the surface of the trypomastigote forms of* Trypanosoma cruzi* and they are expressed in vivo as a ~20-kDa protein during the mammal-derived stages [72–74].

The second mucin family TcSMUG encodes for very small open reading frame containing a putative signal peptide at the N-terminus and a GPI-anchor signal in the C-terminus. This protein family is divided into two groups: small (S) and large (L) according to their encoded mRNA size [67, 71, 75]. The S group encodes for 35–50 kDa mucins N-glycosylated (Gp35/50 mucins) and they are the major acceptors of sialic acid on the parasite surface by parasite trans-sialidases in* T. cruzi*. This S group is found in the epimastigote and metacyclic trypomastigote forms [32, 33]. TcSMUG L group, in contrast, encodes for mucin-type glycoconjugates which are not sialic acid acceptors and they are only present in the surface of the epimastigote stage [34, 76]. Furthermore, depending on the origin of the encoding allele, TcSMUG L products contain one or two additional N-glycosylation signals between the N-terminal region and the threonine-rich region [34].

## 3. Trans-Sialidase Superfamily

**Figure 3:**
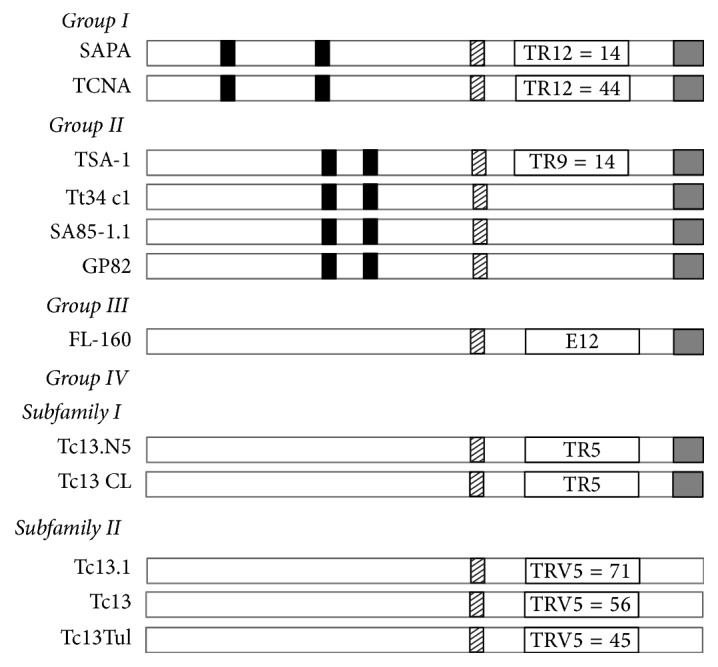
Trans-sialidase (TS) superfamily. Schematic representation of the four different groups of trans-sialidases (TS) from* Trypanosoma cruzi*. Characteristic motifs SXDXGTW and VTVXNVXLYNR for TS are depicted as black and shadowed boxes, respectively. The glycosylphosphatidylinositol- (GPI-) anchor signal in the C-terminus position is shown as grey boxes. Tandem repeats (TR) of 12 amino acid residues [DS_2_AH(S/G)TPSTP(A/V)] are detected in SAPA and TCNA (TR12 inside an open box). Nine amino acid residue repeats [DK_2_ESESGDSE] are identified in TSA-1 (TR9 inside an open box). A characteristic epitope [TPQRKT_2_EDRPQ] is present in FL-160 (E12 inside an open box). The pentapeptide [EPKSA] is found once into subfamily I of Group IV (TR5 inside an open box) whereas, in subfamily II, EPKSA is repeatedly present (TRV5 inside an open box indicating the number of repeats). Image based on Colli and Schenkman [61, 62].

*Trypanosoma cruzi* trans-sialidases (TS) genes are a large superfamily, which includes 1,430 gene members, including 693 pseudogenes [14, 60]. Similar to mucins, TS are distributed along the cell body, flagellum, and flagellar pocket of* T. cruzi* [31, 77]. The TS superfamily is divided into four groups: Groups I to IV (Table 1) [78]. Their sequence similarity and functional properties were used as criteria for classification (Figure 3). Importantly, Group I comprises proteins with trans-sialidase (TS) and/or neuraminidase activities [79]. The TS activity involves the transfer of sialic acid from host glycoconjugates to mainly the parasite mucins present in the plasma membrane of trypomastigotes [80–82]. On the other hand, neuraminidase activity occurs when nonsuitable acceptor molecules for sialic acid are present, and then sialic acid is transferred to water [83]. Trypanosomes are unable to synthesize the monosaccharide sialic acid; they need to scavenge it from the infected host using these TS activities. Therefore the sialylation process in* T. cruzi* is crucial for its viability and propagation into the host [84–87]. Moreover, neuraminidase activity was proposed to be involved in the removal of sialic acid from parasites and/or host-cell molecules which is required for parasite internalization [84, 88]. TS Group I members are as follows: TCNA (neuraminidase), SAPA (shed acute-phase antigen), and TS-epi (Figure 3) [36, 37, 61]. SAPA and TCNA enzymes have active trans-sialidase and neuraminidase activities and are expressed during bloodstream trypomastigote stage [38]. Both enzymes are very close related; when compared they have 84% homology at the aa level. SAPA and TCNA have two main regions: an N-terminal catalytic region and a C-terminal extension, which repeats 12 amino acids (SAPA repeats) in tandem with the consensus sequence: D-S-S-A-H-[S/G]-T-P-S-T-P-[A/V] [89]. SAPA has only 14 tandem repeats compared to 44 for TCNA. The presence of SAPA repeats increases the half-life of the protein in the blood [90]. Both SAPA and TCNA proteins are anchored by glycosylphosphatidylinositol (GPI) to the parasite plasma membrane and can be found in serum from deeply infected mammals [38, 82]. Recently, Lantos and coworkers have shown that domains for mucins and TS are separated by about 150 nm, indicating that mucins do not pass through a TS-rich area for sialylation [31]. Moreover, they proposed a mechanism for the shedding of trans-sialidase into the extracellular space and/or bloodstream via microvesicles, where the phosphatidylinositol-phospholipase-C activity is actually not present in bloodstream trypomastigote stage [31]. TS-epi, the third member of Group I, is an active trans-sialidase expressed in the insect dwelling epimastigote form at the stationary phase and is different from the TS expressed of the blood trypomastigotes. TS-epi lacks SAPA repeats and is not anchored to the membrane by GPI; instead it is predicted that anchoring to the membrane is due to the presence of a transmembrane domain followed by a hydrophilic section in the C-terminus [39]. That last feature may explain why TS-epi is minimally secreted into the medium [91].

TS Group II comprises members of the GP85 surface glycoproteins: ASP-1, ASP-2, TSA-1, Tc85, SA85, GP82, and GP90. They all have been implicated in host-cell attachment and invasion [62, 92–94]. These proteins have complete or degenerate Asp box motifs (SxDxGxTW); the VTVxNVxLYNR motif characteristic of all TS members; and a signal sequence for cleavage/addition of GPI anchor at the C-terminal region (Figure 3) [60, 62, 95]. ASP-1, ASP-2, and TSA-1 are targets of* T. cruzi*-specific CD8^+^ cytotoxic T lymphocytes and they induce strong antibody responses in infected mice and humans [40, 41, 96, 97]. ASP-1 and ASP-2 are amastigote surface proteins, whereas TSA-1 is a trypomastigote surface antigen [40, 41]. SA85 glycoproteins are expressed by amastigote and bloodstream trypomastigote forms. However, only the amastigote form expresses the mannose-binding protein ligand which seems to be involved in the opsonization of the parasite enhancing its infection capability [98–100]. The Tc85 molecule is an 85 kDa glycoprotein and is found abundantly in bloodstream trypomastigotes. Tc85 is identified as a ligand capable of binding to different host receptor molecules (cytokeratin 18, fibronectin, and laminin) located on the cell surface of either monocytes, neutrophils, or fibroblasts [42, 92, 101, 102]. Furthermore, GP82 and GP90 are glycoproteins expressed on the surface of the metacyclic trypomastigote form [46, 103], and they are found mainly at the plasma membrane with opposite roles in mammalian cell invasion [33, 46]. GP82 is able to activate a Ca^2+^ signaling pathway in host cells following parasite adhesion, which is required for* T. cruzi* internalization [101, 104–106]. GP82 binds less efficiently to HeLa cells compared to GP90, but it is capable of triggering the Ca^2+^ signal in that host cell [105]. GP82 is also the signaling receptor that mediates protein tyrosine phosphorylation, which is necessary for host-cell invasion [106]. On the other hand, GP90 is a metacyclic stage-specific glycoprotein defined by its reactivity with monoclonal antibodies 1G7 and 5E7 [46]. GP90 expressed by metacyclic forms lacks any enzymatic activity [47]. GP90 is also present in the mammalian stages of* T. cruzi* (bloodstream trypomastigote and amastigotes stages) and has the antiphagocytic effect mediated by the removal of sugar residues necessary for parasite internalization. This surface glycoprotein appears to have glycosidase activity and downregulates host-cell invasion probably due to the fact that GP90 binds to mammalian cells in a receptor-mediated manner without triggering the Ca^2+^ signal-inducing activity [10, 47].

TS Group III is formed by surface proteins present in mammal-dwelling blood trypomastigotes which include the following: CRP, FL160, CEA, and TESA [48]. These proteins are recognized by sera from patients with Chagas' disease and they are able to inhibit the classical and the alternative pathways of complement activation, which could be a protection from lysis by the host in the trypomastigote form [48, 62, 95, 107–109]. TESA (trypomastigote excretory-secretory antigens) is distributed on the cell surface membrane of* T. cruzi* [107, 110] whereas CRP, FL160, and CEA are flagellum-associated membrane proteins [111–113]. Interestingly, the sequence of FL-160 contains an epitope which molecularly mimics a nervous tissue antigen from the mammalian host [114].

Finally, TS Group IV is composed of genes encoding trypomastigote surface antigens whose biological function is still unknown. This group is included in the TS superfamily because it contains the conserved motif VTVxNVxLYNR, which is shared by all known TS members [10, 37, 54, 61, 115]. However, the B5 peptide from TsTc13 protein, a representative of Group IV, has been shown to be highly antigenic and is present in the infective metacyclic trypomastigote form [49].

## 4. TcGP63 Family

**Figure 4:**
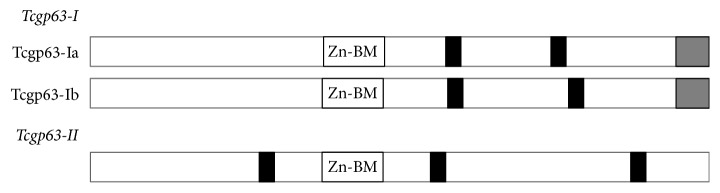
The TcGP63 family. The TcGP63 family consists of cell surface-localized, zinc-dependent metalloproteases also known as* T. cruzi* GP63-like proteins. This family has at least two groups: TcGP63-I and TcGP63-II. TcGP63-I members have two potential N-glycosylation sites, whereas TcGP63-II members have three [50]. The glycosylphosphatidylinositol- (GPI-) anchor signal in the C-terminus position is depicted as grey boxes; it is absent in the TcGP63-II members. Predicted N-glycosylation sites are shown in black boxes. Zn-BM: zinc-binding motifs [VXAHEX_2_HA] associated with metalloprotease activity.

Trypanosomes and* Leishmania* species express a family of cell surface-localized, zinc-dependent metalloproteases, which are also termed as GP63 proteins, major surface proteases, or leishmanolysins. Metallopeptidase activities have been described in trypanosomatids [116–118], but only the so-called GP63 from* Leishmania* spp. has been thoroughly characterized.* Trypanosoma cruzi* possesses GP63-like genes* (TcGP63)* and they are differentially regulated, which suggests its functional importance at multiple stages in the parasite life cycle [52, 119, 120]. The TcGP63 family has at least two groups of proteins: TcGP63-I and TcGP63-II (Figure 4, Table 1) [50]. It has been estimated that* TcGP63-I* has low (5–10) gene copies, whereas* TcGP63-II* has 62 gene copies into the* T. cruzi* genome [50, 51]. The TcGP63-I group is present in the three life-stages of* T. cruzi*. These proteins present metallopeptidase activity and are bound to the protozoan's membrane by a C-terminal glycosylphosphatidylinositol- (GPI-) anchor signal [50]. Two isoforms are known of TcGP63-I in* T. cruzi*: a glycosylated and a nonglycosylated isoform. The 61 kDa glycosylated isoform is present in similar levels in both epimastigote and amastigote forms and is irregularly expressed on the surface membranes (cell body and flagellum) of the epimastigote. The second isoform is a 55 kDa TcGP63 nonglycosylated protein, which is located intracellularly near the kinetoplast and the flagellar pocket of the metacyclic trypomastigote [52]. TcGP63-II does not have GPI-anchor signal; instead its C-terminal sequence is replaced by a charged region containing three Asp and four Arg residues [50, 52].

## 5. Amastin Family

**Figure 5:**
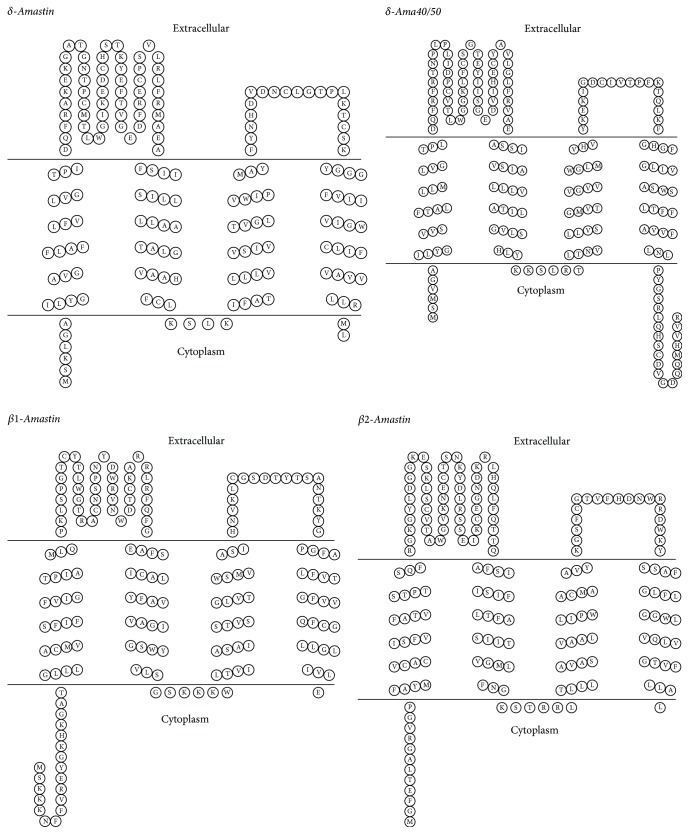
Amastin family. Topological model of subfamilies *β*- and *δ*-amastins from* Trypanosoma cruzi*. Amastins models share four predicted transmembrane helices and two extracellular hydrophilic loops. Although both N- and C-termini are predicted to be facing the cytoplasmic space, their length is variable among them. Topology predictions were performed using the “TOPO2, transmembrane protein display software” (http://www.sacs.ucsf.edu/TOPO2).

The amastin family is a group of transmembrane glycoproteins, which consists of small proteins of about 180 amino acids. Phylogenetic analysis of trypanosomatid amastins has defined four subfamilies named *α*-, *β*-, *γ*-, and *δ*-amastins, with distinct genomic organization as well as patterns of expression during the cell cycle of trypanosomatid [121, 122]. The* Trypanosoma cruzi* genome possesses two distinct subfamilies: *β*- and *δ*-amastins (Table 1), which have predicted the occurrence of four transmembrane regions (Figure 5) [53]. Genes encoding for the *β*1- and *β*2-amastin, belonging to the *β*- subfamily, are localized in the chromosome 32 of* T. cruzi*, whereas *δ*-amastin and *δ*-ama40/50* loci* are found on chromosomes 34 and 26, respectively. *β*1- and *δ*-amastins are clearly located at the cell surface. Interestingly, *β*2-amastin shows a disperse distribution within the cytoplasm in addition to their surface localization [53]. The exact biological function of amastin is still unknown; however, as transmembrane proteins, amastins could play a role in proton or ion traffic across the membrane [123, 124]. Transcript levels of *δ*-amastins are upregulated in amastigotes from different* T. cruzi* strains, while *β*-amastin transcripts are more abundant in epimastigotes than in amastigotes or trypomastigotes; therefore *β*-amastins may be involved in the parasite adaptation to the insect vector [121, 125, 126]. Interestingly, Cruz and coworkers showed that *δ*-amastin plays a crucial role in the differentiation of* T. cruzi*; therefore it is a key molecule responsible for the parasite survival in the intracellular cell stage [127].

## 6. TcTASV Family

TcTASV (Trypomastigote Alanine Serine Valine-rich protein) is a family that comprises 40 members in* Trypanosoma cruzi*. They all have a C- and an N- terminus conserved with a variable central core. This variable core is rich in Ala, Ser, and Val residues, with a conserved Glu-Ala-Pro motif. It also has a high number of Ser and Thr susceptible to glycosylation and a signal peptide and a consensus sequence for the addition of a GPI anchor were predicted, suggesting that this family can be located at the parasite surface and/or be secreted to the milieu [54]. The TcTASV family is conserved across the genomes of* T. cruzi* strains and, to date, no orthologues in other trypanosomatids have been found [55].

TcTASV family was split into three subfamilies: A, B, and C apoproteins, based on their predicted molecular weights (18 kDa, 27 kDa, and 36 kDa, resp.) (Figure 1) [54]. Until now, only subfamilies A and C have been worked thoroughly. Subfamily B has presented experimental hurdles to overcome. Annotated genes identified as TcTASVs are present in 5 chromosomes; almost all annotated subfamily C on the chromosome 24 and a high proportion of subfamily A on the chromosome 16. A peptide entirely conserved in TcTASV-A is present in trypomastigote and amastigote extract. However, only the expression of TcTASV-A in bloodstream trypomastigotes was demonstrated, suggesting that the TcTASV population could undergo developmental regulation [54, 128]. The TcTASV-C subfamily is expressed mainly in the trypomastigote stage as a phosphorylated, heavily glycosylated protein with ca. 60 kDa. TcTASV-C is attached to the parasite surface by a GPI anchor on the cell body and flagellum, which may explain why it is shed spontaneously into the medium and is in contact with the immune system of the host during the course of the natural infection. The superficial localization and secretory nature of TcTASV-C suggest a possible role in the host-parasite interactions [55].

## 7. Mucin-Associated Surface Proteins (MASPs) Family

This family received its name because its members are located in close proximity of* Trypanosoma cruzi* mucins (TcMUC II); they are similar in structure, though not in sequence [129, 130]. MASP members contain N- and C-terminal conserved domains that encode a signal peptide and a GPI-anchor addition site, respectively. MASP is GPI anchored to the membrane and is preferentially expressed during the trypomastigote (bloodstream) stage (Table 1). Moreover, the central region is variable both in length (ranging 176–645 aa) and in sequence; its sequence also contains a large repertoire of repetitive motifs. Single aa residue repetitions are the most common, and those containing glutamic acid are more frequently being around 27% of the total of the identified repetitive motifs. Full-length MASP analysis revealed at least four potential O-glycosylation sites per sequence, 70% of which correspond to threonines [130]. The MASP expression was analyzed throughout the parasite life cycle and it was identified that they are expressed simultaneously in bloodstream trypomastigotes as well as in amastigotes and epimastigotes [45, 128]. MASP molecules are the most abundant antigens found on the surface of the infective trypomastigote stage of* T. cruzi* [130–133]. The overexpression of MASPs in the intracellular parasites prior to the division of the amastigotes located in the plasma membrane suggests that some of the proteins of this extensive family play a major biological role in the survival and multiplication of intracellular amastigotes [130, 134].

## 8. Cruzipain Family

Cruzipains are a papain-like cysteine proteases; cruzipain is expressed as a complex mixture of isoforms in all the* Trypanosoma cruzi* developmental stages (Figure 1) [135]. Despite the fact that cruzipain has high homology with other members of the papain proteases superfamily, this protein has a unique C-terminal region, which is retained in the mature protein [136, 137]. Cruzipains are expressed on all the body surface of epimastigotes and amastigotes. In contrast, on the trypomastigote form, cruzipain has only been present in the flagellar pocket region as well as within the pocket [138]. A specific, irreversible enzyme inhibitors for cruzipain GP57/51 was evaluated in heart muscle cells infected with trypomastigotes and proved to interfere with cell invasion and inhibit* T. cruzi* intracellular replication [139, 140]. The above suggests that cruzipain plays a role in the process of* T. cruzi* internalization into mammalian cells [138–140]. Additionally, cruzipain not only is essential for parasite survival but also generates a strong immune response in infected individuals [138, 141].

## 9. Concluding Remarks

A tangled mechanism is necessary for a “successful” host-pathogen interaction of* Trypanosoma cruzi* with its mammalian or insect host. The availability of the* T. cruzi* genome sequence made it possible to gain new insights into the parasite's biology and allowed the development of new powerful approaches to understand molecular pathogenesis and host-parasite interaction [14]. Proteome analysis was conducted in the different developmental stages of* T. cruz*i; as expected, surface proteins are part of the outstanding proteins which were found differentially expressed among stages [128]. Another proteomic analysis has also been conducted in different organelles, on a specific developmental stage, or under certain stress conditions [8, 142–144]. Additionally, new technologies are now available to facilitate genome editing in* T. cruzi*, such as the Cre-recombinases and the CRISPR-Cas9 system. These genetic manipulation strategies have highly effective efficiency in different organisms and now were successfully adapted to disrupt genes from* T. cruzi* [145–147]. Furthermore the CRISPR-Cas9 system was recently used for endogenous tagging of proteins in* T. cruzi* which proved that this system is not limited to loss-of-function and made the localization/visualization of proteins from inside the parasite possible [148]. These new molecular strategies have now opened a new field of possibilities towards a more comprehensive functional analysis of the parasite biology and can be potentially used to move forward in the study of surface proteins of* T. cruzi*. As we present here, several studies show that surface membrane proteins are crucial for adaptation, differentiation, and survival of the parasite during its life cycle. Notably, some membrane protein families stand out during the host-parasite infection process, which make them potential targets to treat, or even prevent, the infection process. Altogether, these recent advancements can positively increase the current knowledge of host-parasite interactions and will help to accelerate the discovery of effective drugs against the Chagas disease. Despite all the research advances on these protein families on* T. cruzi* membrane, efforts to unravel their structure and function still have a long journey to be undertaken.

## Figures and Tables

**Table 1:** Summary of surface protein families of *Trypanosoma cruzi* and their characteristics.

Protein Family	Group	Members	Host	Parasite stage	References
*Mucin*	TcMUC	TcMUC I	Mammal	Amastigote and bloodstream trypomastigote	[29]
		TcMUC II	Mammal	Amastigote and bloodstream trypomastigote	[30, 31]
		TcMUC III (TSSA)	Mammal	Bloodstream trypomastigote	[30]
	TcSMUG	TcSMUG S	Insect	Epimastigote and metacyclic trypomastigote	[30, 32, 33]
		TcSMUG L	Insect	Epimastigote	[34, 35]

*Trans-sialidase*	TS I	TCNA	Mammal	Bloodstream trypomastigote	[36–38]
		SAPA	Mammal	Bloodstream trypomastigote	[38]
		TS-epi	Insect	Epimastigote	[39]
	TS II	ASP-1 and ASP-2	Mammal	Amastigote	[40, 41]
		TSA-1, Tc85, and SA85	Mammal	Bloodstream trypomastigote	[42–44]
		GP82	Insect	Metacyclic trypomastigote	[45]
		GP90	Insect/mammal	Amastigote, bloodstream and metacyclic trypomastigote	[10, 46, 47]
	TS III	CRP, FL160, CEA, and TESA	Mammal	Bloodstream trypomastigote	[48]
	TS IV	TsTc13	Insect	Metacyclic trypomastigote	[49]

*TcGP63 family*	TcGP63-I	61 kDa glycosylated isoform	Insect/Mammal	Epimastigote and amastigote	[50, 51]
		55 kDa nonglycosylated protein	Insect	Metacyclic trypomastigote	[50, 51]
	TcGP63-II	Two transcripts of 2.6 and 2.8 kb	Insect/mammal	Amastigotes, epimastigote, and bloodstream trypomastigote	[50, 52]

*Amastin family*	*δ*-Amastins	*δ*-Amastin and *δ*-ama40/50	Mammal	Present in all life cycle, up-regulated in amastigotes stage	[53]
	*β*-Amastin	*β*1-Amastin and *β*2-amastin	Insect/mammal	Present in all life cycle, upregulated in epimastigote stage	[53]

*TcTASV family*	TcTASV-A		Mammal	Bloodstream trypomastigote	[54]
	TcTASV-B		Mammal	Bloodstream trypomastigote	[54]
	TcTASV-C		Mammal	Bloodstream trypomastigote	[55]

*MASP family*			Insect/mammal	Amastigote, epimastigote, bloodstream and metacyclic trypomastigote	

*Cruzipain family*	N-cruzipain		Insect	Epimastigote	[56, 57]
	R-cruzipain 1 (cruzain)		Insect/mammal	Epimastigote, bloodstream trypomastigote and amastigote	[58, 59]
	R-cruzipain 2		Mammal	Bloodstream trypomastigote and amastigote	[56]
